# Relative importance of the land‐use composition and intensity for the bird community composition in anthropogenic landscapes

**DOI:** 10.1002/ece3.3534

**Published:** 2017-11-01

**Authors:** Vincent Pellissier, Anne Mimet, Colin Fontaine, Jens‐Christian Svenning, Denis Couvet

**Affiliations:** ^1^ UMR 7204 UPMC‐MNHN‐CNRS CERSP Muséum National d'Histoire Naturelle Paris France; ^2^ Section for EcoInformatics & Biodiversity Department of Bioscience Aarhus University Aarhus Denmark; ^3^ UMR 7533 CNRS‐ Paris 1‐ Paris 7‐ Paris 8‐ Paris 10, LADYSS Paris France; ^4^ Department Computational Landscape Ecology UFZ ‐ Helmholtz Centre for Environmental Research Leipzig Germany; ^5^ Biodiversity Conservation Group German Centre for Integrative Biodiversity Research (iDiv), Halle‐Jena‐Leipzig Leipzig Germany

**Keywords:** agriculture, community structure and functioning, heterogeneity, human appropriation of net primary productivity, human impact, land cover, management, practices, species‐area relationship, species–energy relationship

## Abstract

Humans are changing the biosphere by exerting pressure on land via different land uses with variable intensities. Quantifying the relative importance of the land‐use composition and intensity for communities may provide valuable insights for understanding community dynamics in human‐dominated landscapes. Here, we evaluate the relative importance of the land‐use composition versus land‐use intensity on the bird community structure in the highly human‐dominated region surrounding Paris, France. The land‐use composition was calculated from a land cover map, whereas the land‐use intensity (reverse intensity) was represented by the primary productivity remaining after human appropriation (NPP_remaining_), which was estimated using remote sensing imagery. We used variance partitioning to evaluate the relative importance of the land‐use composition versus intensity for explaining bird community species richness, total abundance, trophic levels, and habitat specialization in urban, farmland, and woodland habitats. The land‐use composition and intensity affected specialization and richness more than trophic levels and abundance. The importance of the land‐use intensity was slightly higher than that of the composition for richness, specialization, and trophic levels in farmland and urban areas, while the land‐use composition was a stronger predictor of abundance. The intensity contributed more to the community indices in anthropogenic habitats (farmland and urban areas) than to those in woodlands. Richness, trophic levels, and specialization in woodlands tended to increase with the NPP_remaining_ value. The heterogeneity of land uses and intensity levels in the landscape consistently promoted species richness but reduced habitat specialization and trophic levels. This study demonstrates the complementarity of NPP_remaining_ to the land‐use composition for understanding community structure in anthropogenic landscapes. Our results show, for the first time, that the productivity remaining after human appropriation is a determinant driver of animal community patterns, independent of the type of land use.

## 1 INTRODUCTION

Within the context of the current biodiversity crisis (Ceballos et al., 2015), it is of vital importance to understand and monitor the impact of human pressures on ecosystems. At least two main spatial dimensions of human pressures can be identified as follows: land‐use types, such as farming or urbanization (Sala, 2000), and the land‐use intensity, such as agricultural intensification and urban density (Erb et al., 2013).

Land use refers to the human use of lands, such as for cropping or pastures, and is linked to practices such as tillage and fertilization. The initiation of more‐intensive uses of land (such as agriculture and urbanization) is usually linked to changes in land cover (such as deforestation), which is defined as the physical coverage of the land, for example, by grass and built‐up areas. Through changes in land cover, land use has large consequences in terms of habitat transitions habitat loss and habitat quality (Newbold et al., 2015). Common data products for the land state (such as CORINE Land Cover for Europe or USGS data for the United States) usually mix information on land use and land cover. Land uses and land covers are widely employed proxies for the mapping and quantification of species habitats and the identification of human pressures on biodiversity (Hudson et al., 2014). For common birds in France, the literature usually recognizes three widespread habitat types based on land use: farmland, forest, and urban habitats (Julliard, Clavel, Devictor, Jiguet, & Couvet, 2006). Hereafter, we refer to land use to describe these combinations of land use and land cover.

**Figure 1:**
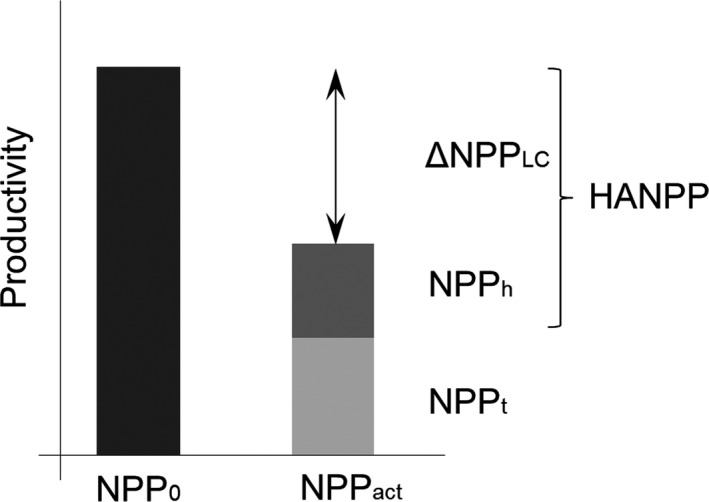
Representation of different NPP measures and how they relate to each other (after Haberl et al., 2007). NPP_potential_: potential NPP, the estimated NPP without any human activity derived from pedo‐climatic conditions; NPP_actual_: actual NPP, the NPP produced annually by the system; NPP_remaining_: NPP remaining after human appropriation, the portion of NPP in the ecosystem after human land cover changes and harvesting; HANPP: Human Appropriation of the NPP, which is divided in two parts: NPP_harvested_ is the NPP harvested through regular human activities via land use, and ΔNPP_LC_ represents changes in NPP due to changes in land cover induced by human activities (such as transitions from forest to farmland or urban areas). Here, we focus on the impacts of NPP_remaining_ and HANPP on bird communities. Because NPP_potential_ is highly homogeneous in the study region, and NPP_remaining_ and HANPP are highly negatively correlated and represent two faces of the same coin

Human Appropriation of Net Primary Productivity (HANPP; Haberl et al., 2007) has been proposed as a measure of land‐use intensity (Erb et al., 2013). Changes in land cover induced by land use can reduce or increase actual Net Primary Productivity (i.e., NPP of the current vegetation, NPP_actual_) compared with the NPP of a pristine ecosystem (potential vegetation productivity, NPP_potential_). Moreover, a substantial fraction of NPP_actual_ is directly removed through the appropriation of agricultural and forestry productivity. This portion is referred to as harvested NPP (NPP_harvested_). Thus, only a fraction of NPP_actual_ remains available for ecosystem processes, referred to as remaining NPP (NPP_remaining_). We note that in some particular cases, highly managed areas produce as much or more than unmanaged areas, especially in open vegetation ecosystems, for example*,* irrigated and mowed grassland or golf courses (Falk, 1980; Wu & Bauer, 2012), but the biomass produced in these areas is usually exported and, hence, no longer available in the ecosystem. NPP_remaining_ is the opposite of HANPP (they sum to NPP_potential_). Consequently, in regions where natural variation in NPP_potential_ is low and the main source of variation in the available productivity is human activity, NPP_remaining_ can be employed as a direct measure of the intensity of human activities (Figure 1). While some might argue that a proper measure of intensity should be expressed as a percentage, and not as a raw productivity value, we used the raw value because (1) it allowed us to derive predictions based on the large corpus of literature on species–energy or species‐productivity relationships; and (2) in our particular case, measures of potential NPP expressed either as raw values or as a percentage of potential NPP were highly correlated (*r* = .99).

In this manuscript, intensity and NPP_remaining_ are synonyms. We alternate between the terms “intensity” (with the advantage of its simplicity) and “NPP_remaining_” when describing ecological processes that involve productivity. Global HANPP ranges from 20% to 40% of NPP_potential_ (Haberl et al., 2007), and its value has doubled over the past century (Krausmann et al., 2013). Despite early concerns that this decrease in available energy for biodiversity might negatively impact biodiversity (Wright, 1990), little is known about the actual effects of NPP_remaining_ on the structure of ecosystems (Haberl et al., 2004, 2005).

### 1.1 Influence of productivity on community indices

The use of NPP_remaining_ as a proxy for the intensity of human pressure primarily relies on the species–energy relationship (Wright, 1983). Productivity and species richness are positively correlated, and this relationship holds true for many taxa and spatial scales (Cusens, Wright, McBride, & Gillman, 2012). Several mechanisms (the More Individuals, More specialization, and More Trophic Levels hypotheses) have been proposed to explain this relationship (Evans, Warren, & Gaston, 2005; Srivastava & Lawton, 1998), all of which imply underlying links between NPP_remaining_ and other community indices, such as total abundance, the length of the trophic chain and specialization.

*The More Individuals* hypothesis states that a positive species–energy relationship is driven by an increase in species abundance, where the underlying assumption is that a more productive area can provide resources to support more individuals, leading to an increase in species richness. An increase in abundance with NPP is commonly observed, though the causal link with species richness has received only mixed support (Currie et al., 2004; Dobson, Sorte, Manne, & Hawkins, 2015; Evans, James, & Gaston, 2006; Evans, Newson, Storch, Greenwood, & Gaston, 2008).

*The More Specialization* hypothesis states that higher energy levels may promote niche‐breadth specialists, where the higher resource partitioning allows coexistence of more species, leading to increased richness. The increased availability of resources and conditions linked to high‐productivity systems is expected to maintain viable populations of specialists (Abrams, 1995; Mason, Irz, Lanoiselée, Mouillot, & Argillier, 2008).

*The More Trophic Levels* hypothesis links increasing richness to an increase in the length of the food chain: higher resource availability allows additive trophic levels, which reduces the size of prey populations, favoring resource partitioning, and allowing more species to coexist (Abrams, 1995; Srivastava & Lawton, 1998).

### 1.2 Land use impacts on community indices

Most of the land use impacts on community indices are a result of trade‐offs occurring between intrahabitat changes (i.e., modification of the area of a given habitat) and interhabitat changes (i.e., modifications of landscape heterogeneity; Allouche, Kalyuzhny, Moreno‐Rueda, Pizarro, & Kadmon, 2012).

Within a habitat type, the habitat area is expected be positively related to richness and abundance through the species‐area relationship (Preston, 1962). The underlying mechanisms proposed to explain the species‐area relationship are similar to those explaining the species–energy relationship, simply because the habitat area is a proxy of available resources (Wright, 1983). Regarding productivity, large areas of habitat have been shown to favor specialist species (Matthews, Cottee‐Jones, & Whittaker, 2014). The habitat area has also been shown to have a positive effect on the abundance of species at the end of the trophic chain (Davies, Margules, & Lawrence, 2000). Because higher‐level consumers have greater energetic needs, they also exhibit a smaller population size, increasing their risk of extirpation as the habitat area decreases (Holt, Lawton, Polis, & Martinez, 1999).

Mechanisms differ at the landscape scale when considering changes in the area of several habitats simultaneously, where species richness is often positively correlated with the heterogeneity of land uses and habitats (Martins, Proença, & Pereira, 2014; Stein, Gerstner, & Kreft, 2014). Land‐use heterogeneity enables species with different ecological requirements to colonize and coexist in a landscape (Bennett, Radford, & Haslem, 2006). However, because heterogeneity is often negatively correlated with an increasing area of dominant land uses in a landscape, the corollary of this statement is that richness usually decreases as the proportion the dominant land uses increases (Allouche et al., 2012). This increase in richness with moderate heterogeneity results from an increase of habitat generalist species, which are favored by heterogeneous landscapes (Mimet, Houet, Julliard, & Simon, 2013), and a smaller decrease in specialists related to habitat loss (Allouche et al., 2012).

### 1.3 Objectives of the study

Only two studies have explored the species–energy relationship accounting for human impacts due to harvesting and land cover changes (i.e., NPP_remaining_; Haberl et al., 2005; Mouchet et al., 2015). Neither of these studies explored the response of other community indices that are theoretically linked to richness. These studies did not account for the various land‐use types in the landscape (Haberl et al., 2005) or were performed at a coarse scale and resolution (Mouchet et al., 2015). As a consequence, the relative importance of the land‐use composition (type) versus NPP_remaining_ in explaining community structure remains largely unknown.

Here, we aim to (1) disentangle the relative influence of the land‐use composition (type) and intensity (i.e., NPP_remaining_) on bird community structure; and (2) test the existence of the species–energy relationship in human‐dominated landscapes, accounting for confounding factors of the land‐use composition.

We conducted our study in the region of Paris in France, using Landsat remote‐sensing and bird data from the French Breeding Bird Survey. This highly human‐dominated region is modified by both intensive agriculture and urbanization (Mimet et al., 2013) and exhibits little natural variation in potential vegetation productivity (Haberl et al., 2007). The potential NPP, as computed using the Lund‐Jena‐Potsdam Dynamic Global Vegetation Model (Sitch et al., 2003) by Haberl et al. (2007), ranges between 641 and 663 gC year^−1^ m^−2^ (mean ± *SD* = 654 ± 5 gC year^−1^ m^−2^). Therefore, our results can be interpreted as indicating the impacts of the available productivity or the intensity of human productivity appropriation on species communities.

Because the effect of productivity on species richness is known to be habitat dependent (Verschuyl, Hansen, McWethy, Sallabanks, & Hutto, 2008), we address these questions in local communities belonging to three different habitat types that differ in productivity (woodland, farmland, and urban habitats). We tested the following predictions:

#### 1.3.1 Predictions for NPP_remaining_ (intensity)

We expect richness to increase with NPP_remaining_. Based on the three previously noted hypotheses regarding the underlying mechanisms of the species–energy relationship, we expect abundance, trophic levels, and habitat specialization to increase with NPP_remaining_ in every habitat. We expect the slope to be steeper in highly anthropogenic habitats (farmland and urban habitats, in which NPP_remaining_ is low and may be limiting) than in habitats with relatively low anthropogenicity (woodlands). We also expect heterogeneity of NPP_remaining_ to increase species richness and reduce habitat specialization.

#### 1.3.2 Predictions for the land‐use composition

Because of the trade‐off between the amount of habitat and heterogeneity at the landscape scale, we expect land‐use heterogeneity, and not the amount of habitat, to increase richness. We expect habitat specialization to decrease with land‐use heterogeneity. Because of the mechanisms underlying the species‐area relationship, we expect trophic levels and habitat specialization in a given habitat to increase with the amount of that habitat in the landscape.

## 2 MATERIALS AND METHODS

### 2.1 Types of human pressures: land‐use variables

The studied area was the Ile‐de‐France region, which covers 12,011 km^2^ and includes Paris. This region is the most populated region in France, with almost 12 million people, at a density of 996 inhabitants per km^2^ in 2013. It is also one of the main agricultural regions and presents highly productive areas (45% of the region is farmland). Still, 23% of the region is covered by forest. The Institute of Urban Planning and Development in the Paris Region (IAU) provided the land‐use database for 2003, with a resolution of 25 m. The information provided by the land‐use database was simplified by grouping its 83 land‐use classes into five land‐use classes, as follows:

1. Farmland areas: areas devoted to agricultural activities: farming or, very rarely, pasture.
2. Urban areas and traffic and train infrastructure: built areas, urban parks, and gardens, building grounds (e.g., swimming pools), roads, railroads, and parking.
3. Woodlands: natural woodlands, forests and poplar groves, forest clearings.
4. Water and wetlands: rivers, other bodies of water and wetlands.
5. Other/Open areas: nonagricultural grasslands, quarries.

### 2.2 Human pressure intensity: NPP remaining after appropriation

NPP_remaining_ is calculated as the amount of NPP remaining in a given locality after human needs have been fulfilled; thus, it is a combination of the appropriation and productivity changes due to land use (Haberl et al., 2007). NPP_remaining_ is the difference between the actual NPP (NPP_actual_, i.e.*,* actual NPP measured in the prevailing vegetation), and the NPP that is harvested or destroyed in woodlands or farmlands (NPP_harvested_; Figure 1). To achieve a fine spatial resolution (30 m pixels), we chose to calculate NPP_remaining_ on a slightly different basis from that employed by Haberl et al. (2007). Instead of inferring the NPP_actual_ value from yield measurements, we calculated the NPP_remaining_ values using satellite imagery and inferred the NPP_harvested_ values from harvest factors found in the literature. A major advantage is the possibility of calculating NPP_remaining_ for highly urbanized pixels, which otherwise would have been considered nonproductive (Figure 1).

#### 2.2.1 Actual NPP: NPP_actual_

NPP_actual_ is the NPP produced by the ecosystem and is estimated using remote sensing data. NPP_actual_ incorporates changes in productivity induced by human activity, particularly urbanization and agriculture. NPP_actual_ was assessed for the year 2003 using monthly NPP values. These monthly values were calculated over the entire Paris region in 30 × 30 m pixels using the CASA‐NASA (Carnegie‐Stanford‐NASA) approach for estimating aboveground NPP, as employed by (Potter, Gross, Genovese, & Smith, 2007). The basis of this approach is the use of a single satellite image at the approximate peak of photosynthesis and the use of other time variants to assess monthly NPP, as expressed in Equation (1):

(1) NPP=0.39×EVI×Sr×T×W,

where EVI is the Enhanced Vegetation Index. EVI was determined using a Landsat 7 image from May 29, 2003 and calculated according to Equation (2):

(2) $$\text{EVI}\;=\;2.5\;\times \;\left(\frac{\rho \text{NIR}\;-\;\rho \text{RED}}{\rho \text{NIR}\;+\;6\;\times \;\rho \text{RED}\;-\;7.5\;\times \;\rho \text{BLUE}\;+\;1}\right),$$

where ρNIR, ρRED, and ρBLUE represent the surface reflectance acquired in the near‐infrared, red, and blue regions, respectively.

The Sr term in Equation (1) denotes the land surface radiation balance (in W/m^2^) as calculated by Ryu, Kang, Moon, and Kim (2008). This radiation balance was calculated using moderate‐resolution imaging spectroradiometer (MODIS) products, and the values needed to calculate Sr were derived from monthly MODIS products. The MOD08 product (Atmosphere) was employed to derive the optical density, ozone levels, water levels, and atmospheric dew point for each month; MOD43 (Albedo product) was used to calculate monthly values of the surface albedo; and MOD11 (emissivity and temperature) was employed to derive monthly land emissivity and surface temperature.

The T term in Equation (1) is a temperature stress scalar calculated as the departure of the mean temperature from an optimal temperature for vegetation growth. This optimal temperature was uniformly set at 22°C. The water stress scalar (*W*) was considered the water deficit for the growth of vegetation and was calculated the difference between water needs (potential evapotranspiration, PET) and available water (actual evapotranspiration, AET). Both evapotranspiration terms were calculated using the Thornthwaite simple bucket water balance model (Thornthwaite, 1948). The *T* and *W* terms were calculated using gridded, monthly climatic variables that are freely available from the European Climate Assessment & Dataset project (ECA, 2013).

A part of NPP is transmitted to the ecosystem during plant growth (via weeds and herbivory) before the harvest and remains available in the system. To account for this portion of NPP, we applied a loss factor obtained by Haberl et al. (2007) for Western Europe. This corrected measure of NPP was defined as NPP_actual_.

#### 2.2.2 NPP remaining after appropriation: NPP_remaining_

NPP_remaining_ is the productivity remaining after appropriation. To calculate NPP_remaining_ for the study area, we first evaluated the land use in each pixel. For farmland areas (croplands, orchards, and permanent pastures), we evaluated NPP_actual_, the proportion of NPP_actual_ that was cropped, the exported residues and the residues left on the ground. NPP_remaining_ was, therefore, the sum of the NPP losses and remaining crop residues. The NPP_actual_ components of farmlands were calculated using three harvest factors (for croplands, pastures, and orchards) and recovery rates for Western Europe obtained from the literature by Haberl et al. (2007). Harvest factors determine the mass of vegetation residues that remain in place after harvest and mainly depend on the degree of mechanization. Agricultural activity in the Ile‐de‐France region is highly homogeneous, being strongly dominated by large farms applying intensive conventional practices in cereal fields (http://agreste.agriculture.gouv.fr). The homogeneity of the agricultural practices in the studied region justifies the use of a unique harvest factor across sites. However, as we employed a single date for NPP_remaining_ estimation, we could not blindly apply a harvest factor for forests. Forests are harvested at long intervals, ranging from decades to centuries. Applying the same harvest factor over every forested area would have strongly over‐ or under‐estimated the actual harvest depending on recent local actions.

Thus, even though we employed harvest factors defined at a continental scale, which might appear somewhat coarse, and used no harvest factor for forests, we are confident that our computation of NPP_remaining_ still represents a valid measure of the land‐use intensity. Indeed, measuring NPP_actual_ using remote sensing partly integrates the management measures applied on the ground: if one considers that management changes productivity, it will be reflected in photosynthetic activity and, thus, in satellite imagery, even when only a single image is used.

### 2.3 Description of bird communities

#### 2.3.1 Bird survey data

**Figure 2:**
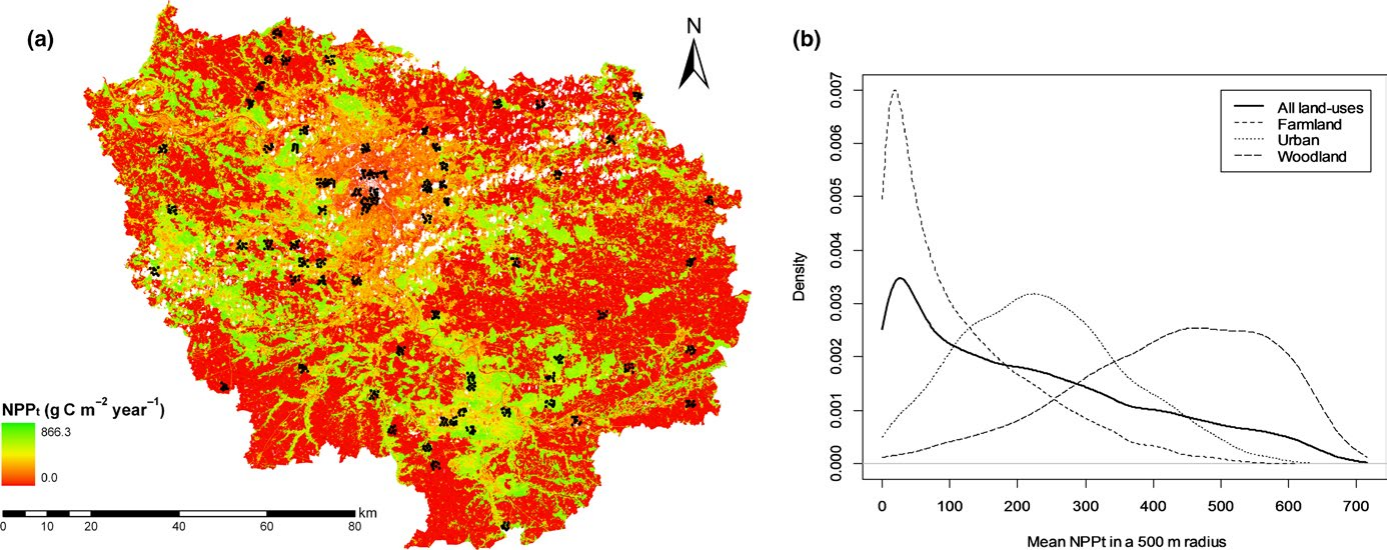
NPP_remaining_ (NPP remaining after human appropriation) (a) in the Paris region of France. The black dots represent the breeding bird survey plots used in our analysis. (b) Kernel density estimation of NPP_remaining_ across the entire region and for each land use

Common bird species are considered good indicators of disturbance and community alterations, particularly if intermediate‐to‐high commonness and guild representation are considered (Koch, Drever, & Martin, 2011). The bird data were derived from the French Breeding Bird Survey, which is based on a standardized monitoring methodology (Jiguet, Devictor, Julliard, & Couvet, 2012). Censuses of breeding birds were performed each spring at randomly selected sites in continental France (Figure 2a) by skilled observers. A total of 520 count points distributed among 58 survey squares of 2 × 2 km were surveyed at least twice between 2001 and 2009. The points within each square were evenly distributed and were located at least 300 m apart. At each point, every individual that was observed or heard during a period of exactly 5 min was recorded. Each count point was monitored twice in the spring (before and after 8 May) to record both early and late breeders, with an interval of 4–6 weeks between the two surveys, and the maximum value from these two monitoring was retained. The local habitat at each point was defined as the land use covering more than 50% of a 100 m radius around the point (Mimet et al., 2013). Thus, each point was classified as belonging to one of the farmland, woodland, or urban habitats. In the final dataset, 135 points were classified as urban, 143 as farmland, and 132 as woodland, leading to a final dataset of 410 points.

#### 2.3.2 Community indices

The following four community indices were selected to derive a synthetic and informative description of the community structure at the count‐point level: (1) species richness; (2) total abundance, calculated as the sum of all observed individuals independent of species identity; (3) the Community Trophic Index (CTI), a measure of the average trophic level of a local bird community (Mouysset, Doyen, & Jiguet, 2012) weighted by species abundance; and (4) the Community Specialization Index (CSI), a measure of the specialization level of the community (Julliard et al., 2006).

To calculate the CTI, we estimated the proportion of plant, invertebrate, and vertebrate items in the diet of each bird species. The species trophic index was defined as the exponential of the weighted mean of these values using weights of 1, 2, and 3 for plant, invertebrate, and vertebrate items, respectively. As a result, an increase in the CTI tends to indicate an increase in the abundance of high trophic levels. The trophic index was calculated using diet data extracted for each species from the Birds of the Western Palearctic Interactive database (BWPi, 2006).

The CSI is the averaged value of the Species Specialization Index (SSI) for all species found in a community (Julliard et al., 2006) weighted by abundances. The SSI is based on the number of habitats in which a species has been observed; for this purpose, data from the French Breeding Bird Survey for the French Atlantic biogeographic region, where our study area is located, were used in this case. According to the definition of the European Bird Indicator adapted for France (http://vigienature.mnhn.fr/page/le-suivi-temporel-des-oiseaux-communs-stoc), when considering three main habitats (woodland, urban, and farmland), a species is defined as a specialist of one habitat when it is twice as abundant in that habitat as in the two other habitats, whereas a species is defined as a generalist when it is roughly equally distributed in the three habitats.

We calculated a global CSI, by integrating all species in the community, as well as a habitat CSI, by integrating only the generalist species, and the specialists of the focal habitats (i.e., excluding the specialists for the other habitats). Thus, we calculated three habitat‐specific CSIs for the communities observed in the urban, woodland, and farmland areas. These habitat‐specific CSIs provide information for the portion of the community that is strongly linked to the target habitat, whereas the global CSI provides composition information for habitat specialists throughout the entire community and is related to the importance of the other land uses in explaining community patterns.

#### 2.3.3 Land‐use composition and intensity

The land‐use composition and intensity variables were assessed in circles with a 500 m radius around each count point. This scale has been found to be suitable for multispecies studies of bird‐landscape relationships among woodland species (Caprio, Ellena, & Rolando, 2008) and farmland species (Smith, Fahrig, & Francis, 2011). The land‐use intensity and the heterogeneity of the land‐use intensity were measured as the average and standard deviation of the NPP_remaining_, respectively, in each landscape defined by the 500 m radius circle. The land‐use composition was described by the proportions of farmland, woodland, and urban areas. Land‐use heterogeneity was described by the total number of different land uses. The richness of land uses was employed as a heterogeneity index because it is less inherently linked to land‐use proportions than a community index based on these proportions (such as the Shannon index), although the richness and Shannon indices were highly correlated (Pearson correlation of .69). Furthermore, our hypotheses regarding the underlying heterogeneity suggested that high land use richness increases the attractiveness of the landscape for generalist species, whereas the habitat area promotes specialist species. Diversity measures assign a higher importance to the land‐use area than the number of land uses; therefore, these measures are not appropriate for addressing our hypothesis.

### 2.4 Statistical analyses

To disentangle the effect of the land‐use composition and intensity on bird communities, we employed two different analyses based on the same model (Equation (3)): (1) We estimated the sign and amplitude of the effect of each individual variable on our community indices; and (2) we estimated the relative contributions of each of the land‐use composition and intensity variables, which allowed us to determine which variable type—composition or intensity—best explained the variability of biodiversity.

As mentioned earlier in this study, a portion of NPP_remaining_ concerns productivity linked to the type of land cover, driven by land use (ΔNPP_LC_, Figure 1). This same part of productivity is contained within the type of land use (shared information), for example, reflecting the productivity loss linked to a forest turned into a pasture. To correctly disentangle the contributions of the land‐use composition (type) and of the intensity (NPP_remaining_), we had to ensure that this shared information was only attributed to NPP_remaining_ and was removed from the land‐use composition. Leaving this information in the composition variables indeed means leaving a land‐use intensity component in those variables, whereas our goal was to disentangle composition from intensity.

We, therefore, devised a residual regression model (Equation (3)) in which the composition variables were replaced by the residuals of regressions of NPP_remaining_ on land‐use proportion variables (Equations (4) and (5)). This series of models is referred to as the residual composition models.

(3) $$\begin{array}{cc} Y & ∼\text{Res(PC1)}\;+\;\text{Res(PC2)}\;+\;\text{LU div}\\ & \;+\;\text{mean}\;{\text{NPP}}_{\text{remaining}}\;+\;\text{SD}\;{\text{NPP}}_{\text{remaining}}\;+\;\text{sunset}\\ & \;+\;\text{Julian}\_\text{date}\;+\;(1|\text{site}\_\text{id})\;+\;(1|\text{habitat}), \end{array}$$

(4) $$\text{PC1}∼\text{mean}\;{\text{NPP}}_{\text{remaining}},$$

(5) $$\text{PC2}∼\text{mean}\;{\text{NPP}}_{\text{remaining}},$$

where *Y* represents a community index (i.e.*,* species richness, total abundance, CTI, and CSI). All of the dependent variables were log‐transformed prior the analyses to ensure a normal distribution of the variables. The right‐hand side of the model comprises several elements, detailed below. Res(PC1) and Res(PC2) are the residuals of PC1 and PC2 extracted from Equations (4) and (5) (Linear Model). PC1 and PC2 are the coordinates of a Principal Component Analysis (PCA) carried out on the landscape composition data (percentage of urban, farmland, and woodland area in the 500 m radius). As the sum of the composition data amount to 100% for each site, including these data in a model might induce multicollinearity. Using coordinates from a PCA ensured that these two variables were uncorrelated. The first two axes of the PCA explained 52.8% and 47% of the variability, respectively. PC1 represented an axis of increasing urban areas and decreasing woodland and farmland areas. PC2 represented an axis of increasing farmland areas and decreasing woodland areas.

LU div represents the richness of land use in the 500 m radius and is computed as the number of different land uses. Mean NPP_remaining_ and *SD* NPP_remaining_ represent metrics of the land‐use intensity and are, respectively, the average and standard deviation of NPP_remaining_ in the 500 m radius. We standardized the independent variables to make the coefficients comparable. Given that our sampling design consisted of repeated measures in time, we added the sampling point identity as a random effect, to avoid any pseudo‐replication issues. Because the temporal trends are not a measure of interest in our case, we did not add the sampling years as a cross‐classified random effect. To account for detectability issues, we added the Julian day and the time after sunset to the model as fixed variables as well as the local habitat as a random variable (see Appendix 1 for the justification of these variables to correct for detectability).

The model (Equation (3)) was run as a Linear Mixed‐Effect Model (LMER, lme4 package, Bates et al., 2015).

(1) To measure the sign and amplitude of the effect of each composition and intensity variable, we used a model‐averaging procedure (delta‐AIC < 4; MuMIn package; Barton, 2013; See Appendix 2for details on the procedure). (2) To measure the contribution of each composition and intensity variable, we applied a hierarchical variance partitioning procedure to the full model described in Equation (3) (hier.part package; Walsh & Mac Nally, 2013). The hierarchical variance partitioning procedure basically determines the independent contributions of variables to explain the variability of the dependent variable, once accounting for detectability in our case. We employed the marginal *R* ^2^ defined for mixed linear models by Nakagawa and Schielzeth (2013), as the marginal *R* ^2^ is defined by these authors as the variance explained only by the fixed part of the model.

We ran all of the above analyses on four datasets: (1) the entire dataset and subsets for the (2) urban, (3) farmland, and (4) woodland habitats (hereafter, All‐habitats, Urban, Farmland, and Woodland models). Each point was assigned to a dataset depending on its local habitat (i.e., the habitat covering more than 50% of its area within a 100 m radius). The random effect on the local habitat was removed for the last three models. We used the global CSI for the All‐habitats dataset and the Urban, Farmland, and Woodland CSIs for the corresponding datasets. Significant spatial autocorrelations were not observed in the residuals of the models; therefore, we did not explicitly correct our models for spatial autocorrelations.

Collinearity was evaluated in all of the models using the Variance Inflation Factor (VIF). The VIF was under 10 for all subset models (Dormann et al., 2013).

To control for the importance of the shared information between NPP_remaining_ and the land‐use composition, we also ran all analyses using two slightly different model expressions, in which the composition variables included the part of intensity related to the land‐use composition (modifications of Equation (3)). First, in a “classic” series of models, we employed the initial composition variables (PC1 and PC2) instead of their residuals. In this series of models, the intensity linked to the land cover is shared by the models between the two types of variables. Second, in a “Residual NPP_remaining_” series of models, we used the residual of the NPP_remaining_ regressed with PC1 and PC2 (mean NPP_remaining_ ~ PC1 + PC2), instead of the NPP_remaining_ value. This series of models provides information about the importance of the portion of intensity independent from the composition in land uses.

## 3 RESULTS

The distribution of the NPP_remaining_ values (expressed as a density function, Figure 2b) varied substantially among the land uses. The woodland presented a high density, of approximately 600 gC m^−2^ year^−1^, whereas farmland presented a density of 100 gC m^−2^ year^−1^, and urban areas presented a density of 200 gC m^−2^ year^−1^.

**Figure 3:**
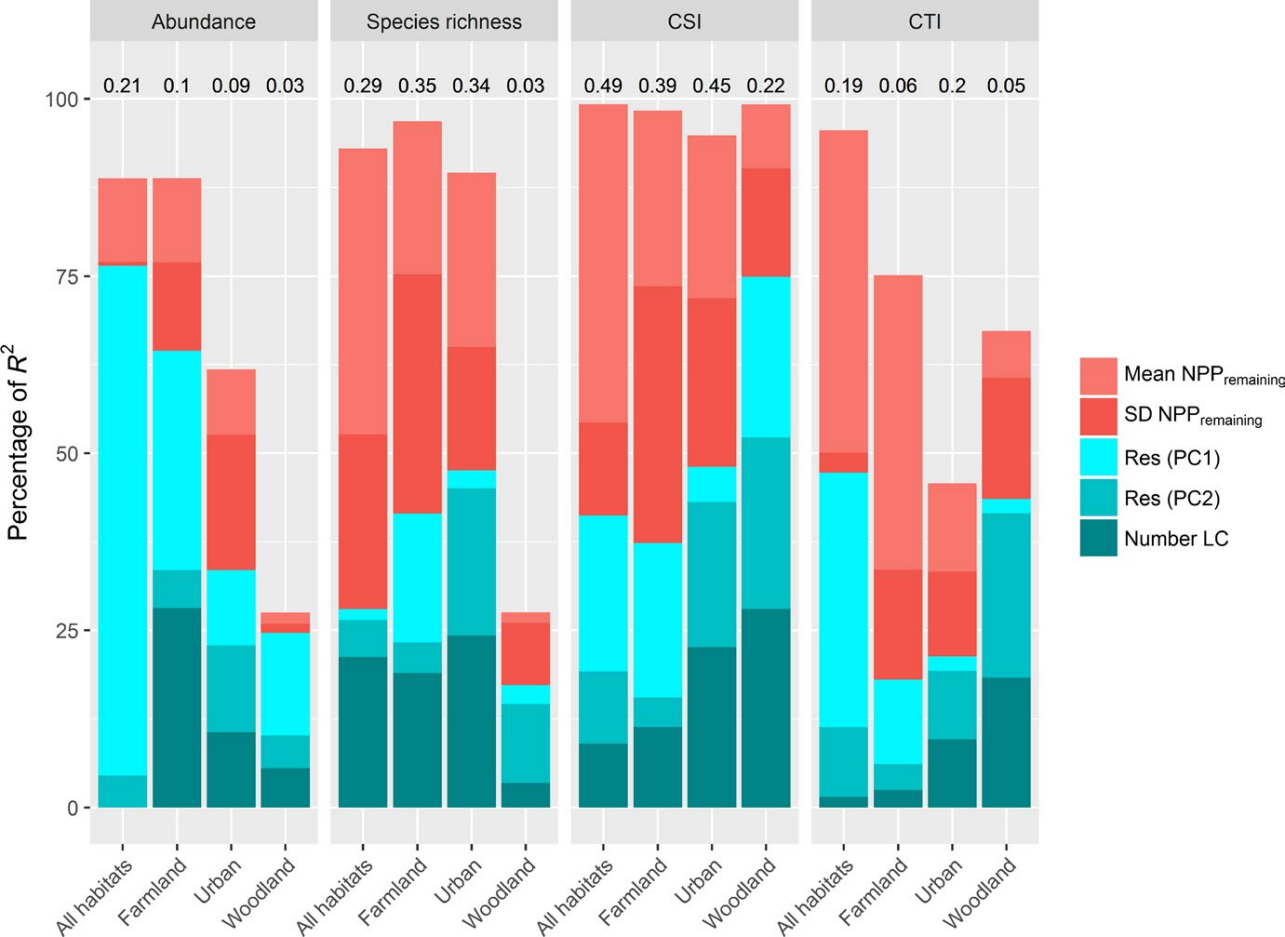
Results of the hierarchical variance partitioning analyses from the “Residual composition” series of models. The bars show the contribution of each composition and intensity variable (Mean NPP_remaining_: average value of NPP_remaining_; SD NPP_remaining_: Standard deviation of NPP_remaining_; Res(PC1) and Res(PC2): residuals of the linear model PC1/PC2~Mean NPPremaining, with PC1/PC2 being the first two axes of a PCA carried out on land cover composition data; Number LC: land cover diversity to the four indices of community structure (Total abundance, Species richness, Community Specialization Index, Community Trophic Index), for the All‐habitats model and the three habitat‐specific models (red shades for the intensity‐related variables and green shades for the composition variables). The values are expressed as the percentage of the explained variance, computed as a coefficient of determination: *R* ^2^ as defined by Nakagawa and Schielzeth (2013). The value of *R* ^2^ for the full model is provided above each bar

The explanatory power of the models of the “Residual composition”, “Classic,” and “Residuals NPP_remaining_” series was similar (Figure 3 and Appendices 3 and 4). Logically, intensity variables were more important in explaining the community indices in the “Residual composition” series, followed by the “Classic” series and the “Residual NPP_remaining_” series. As explained in the methods, we focus on the results of the “Residual composition” series of models hereafter. The results of the two other series are available in Appendices 3 and 4.

The models generally presented a higher explanatory power for habitat specialization (pseudo *R* ^2^ from 0.22 to 0.49) and species richness (pseudo *R* ^2^ from 0.03 to 0.35) than for the trophic index (pseudo *R* ^2^ from 0.05 to 0.19) and total abundance (pseudo *R* ^2^ from 0.03 to 0.21; Figure 3). The trends were generally consistent among the habitats, and the differences between habitats usually consisted of strong variations in the values of the coefficients (Table 1; Figure 3).

**Table 1:** Results of the model‐averaging procedures and the variance partitioning analyses

		Res(PC1): Woodland and farmland to urban	Res(PC2): Farmland to woodland	Land cover number	Mean NPP_remaining_	*SD* NPP_remaining_	Julian days	Minutes after sunset
Abundance	All habitats	**0.2 ± 0.02** *** **(1)**	−0.02 ± 0.03 (0.31)	0.02 ± 0.02 (0.3)	−**0.08 ± 0.02** *** **(0.91)**	**0.04 ± 0.02** † **(0.73)**	**0.05 ± 0.01** *** **(1)**	−**0.05 ± 0.01** *** **(1)**
Abundance	Farmland	**0.08 ± 0.04** † **(0.71)**	−0.04 ± 0.04 (0.39)	**0.09 ± 0.04** * **(0.85)**	0.04 ± 0.05 (0.39)	0.04 ± 0.04 (0.43)	**0.06 ± 0.02** ** **(0.98)**	0 ± 0.03 (0.27)
Abundance	Woodland	0.02 ± 0.02 (0.27)	0.01 ± 0.03 (0.21)	0.02 ± 0.02 (0.22)	−0.01 ± 0.02 (0.19)	0.01 ± 0.02 (0.19)	**0.02 ± 0.01** † **(0.79)**	−**0.05 ± 0.02** ** **(1)**
Abundance	Urban	0.05 ± 0.04 (0.45)	−0.05 ± 0.04 (0.38)	−0.04 ± 0.04 (0.33)	−0.04 ± 0.04 (0.34)	−0.06 ± 0.04 (0.47)	**0.05 ± 0.02** ** **(1)**	−**0.07 ± 0.02** ** **(1)**
Species richness	All habitats	−0.02 ± 0.01 (0.39)	−**0.05 ± 0.01** ** **(1)**	**0.07 ± 0.01** *** **(1)**	**0.14 ± 0.01** *** **(1)**	**0.08 ± 0.01** *** **(1)**	0.01 ± 0.01 (0.52)	−**0.05 ± 0.01** *** **(1)**
Species richness	Farmland	**0.05 ± 0.03** * **(0.74)**	−0.04 ± 0.03 (0.52)	**0.07 ± 0.03** ** **(0.97)**	0.02 ± 0.03 (0.36)	**0.13 ± 0.04** ** **(1)**	0.01 ± 0.01 (0.42)	−**0.03 ± 0.02** * **(0.76)**
Species richness	Woodland	−0.01 ± 0.02 (0.2)	0.01 ± 0.02 (0.23)	0.01 ± 0.02 (0.2)	−0.01 ± 0.02 (0.2)	0.02 ± 0.02 (0.27)	0 ± 0.01 (0.2)	−**0.04 ± 0.01** ** **(1)**
Species richness	Urban	0.04 ± 0.04 (0.38)	0.01 ± 0.03 (0.22)	**0.09 ± 0.03** ** **(1)**	**0.09 ± 0.04** * **(0.86)**	0.05 ± 0.04 (0.47)	0.01 ± 0.01 (0.31)	−**0.08 ± 0.02** *** **(1)**
CTI	All habitats	−**0.03 ± 0.01** *** **(1)**	−**0.01 ± 0** † **(0.67)**	0.01 ± 0 (0.55)	**0.03 ± 0** *** **(1)**	0.01 ± 0 (0.56)	**0.01 ± 0** ** **(1)**	**0.01 ± 0** * **(0.91)**
CTI	Farmland	−**0.01 ± 0.01** * **(0.81)**	−0.01 ± 0.01 (0.53)	0 ± 0.01 (0.3)	**0.02 ± 0.01** ** **(0.97)**	0 ± 0.01 (0.31)	−0.01 ± 0 (0.44)	**0.01 ± 0.01** * **(0.83)**
CTI	Woodland	0 ± 0 (0.22)	−**0.01 ± 0** † **(0.75)**	−0.01 ± 0 (0.53)	0.01 ± 0.01 (0.47)	−0.01 ± 0.01 (0.48)	−**0.01 ± 0** ** **(1)**	0 ± 0 (0.31)
CTI	Urban	−0.01 ± 0.01 (0.4)	0 ± 0.01 (0.3)	0.01 ± 0.01 (0.61)	**0.02 ± 0.01** † **(0.84)**	0.01 ± 0.01 (0.52)	**0.04 ± 0** *** **(1)**	0 ± 0.01 (0.28)
CSI	All habitats	**0.12 ± 0.01** *** **(1)**	**0.05 ± 0.01** *** **(1)**	−**0.06 ± 0.01** *** **(1)**	−**0.19 ± 0.01** *** **(1)**	−**0.07 ± 0.01** *** **(1)**	**0.02 ± 0.01** * **(0.89)**	**0.02 ± 0.01** † **(0.68)**
CSI	Farmland	−**0.09 ± 0.03** ** **(0.97)**	0.03 ± 0.02 (0.5)	−**0.04 ± 0.02** † **(0.59)**	0 ± 0.03 (0.3)	−**0.14 ± 0.04** *** **(1)**	−**0.03 ± 0.01** * **(0.9)**	0.02 ± 0.02 (0.4)
CSI	Woodland	−**0.03 ± 0.01** ** **(1)**	−**0.04 ± 0.01** ** **(1)**	−**0.03 ± 0.01** * **(0.89)**	**0.04 ± 0.02** * **(1)**	0 ± 0.01 (0.19)	−0.01 ± 0.01 (0.42)	0 ± 0.01 (0.19)
CSI	Urban	0.02 ± 0.02 (0.37)	0.02 ± 0.02 (0.34)	−**0.06 ± 0.02** ** **(1)**	−**0.06 ± 0.02** ** **(1)**	−**0.07 ± 0.03** ** **(1)**	**0.03 ± 0.01** ** **(1)**	**0.04 ± 0.02** ** **(1)**

The importance value is provided in parentheses. An importance of 0.5 corresponds to the selection of the variable in 50% of the best models (delta‐AIC = 4), and an importance of 1 corresponds to the selection of the variable in 100% of the best models. Bold values indicate p < 0.1.

^†^ *p* < .1; *p < .05; **p < .01; ***p < .001.

### 3.1 Relative importance of composition and intensity

The importance of the composition and intensity variables was usually comparable. The clearest patterns were a lower contribution of intensity variables (1) for abundance (and all habitats) than for the other community indices; and (2) for woodland habitats (and all indices) than for the other habitats (Figure 3). In the other cases, intensity variables were usually slightly more important than composition variables.

### 3.2 Individual effects of composition and intensity variables

The majority of the observed patterns were close to the predictions, although some responses were unexpected (Table 1, Appendices 5, 6, 7, and 8).

**Figure 4:**
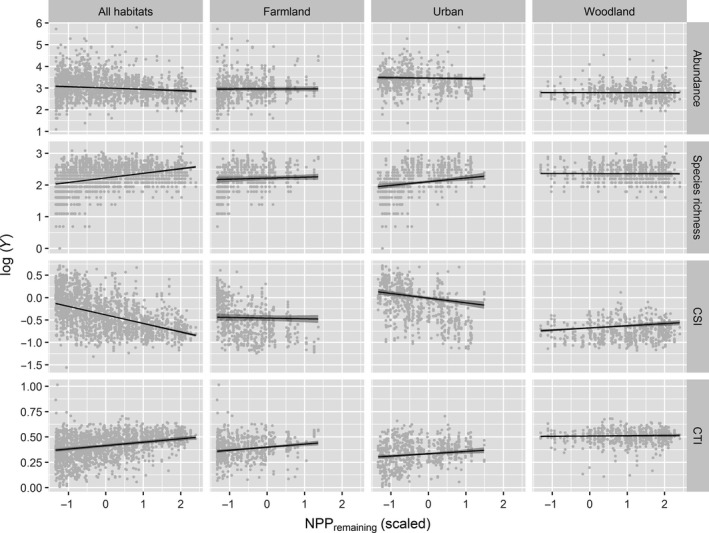
Variations in the four community indices (Abundance, Richness, Trophic level (CTI) and habitat specialization [CSI]) with NPP_remaining_ across all habitat types and for each type (Farmland, Urban, and Woodland) separately. The dots represent the points, and the lines represent the relationships obtained by the models. The error on either side of the lines corresponds to the standard error

Predictions on NPP_remaining_: We expected richness, abundance, trophic levels, and specialization to increase with NPP_remaining_. We expected these responses to be stronger in anthropogenic habitats. We also expected heterogeneity of NPP_remaining_ to increase species richness and reduce habitat specialization. A positive relationship between richness and NPP_remaining_ was indeed observed, but only in the All‐habitats and Urban models (Figure 4). A similar response was observed for the trophic level in the All‐habitats and Farmland models. Contrary to expectations, we detected a negative response of abundance to NPP_remaining_ in the All‐habitats model. Habitat specialization increased with NPP_remaining_ in the Woodland model but unexpectedly decreased in the All‐habitats and Urban models. Richness increased with NPP_remaining_ heterogeneity in the All‐habitats and Farmland models, and specialization decreased with NPP_remaining_ heterogeneity in all of the habitats except for woodlands.

Predictions for the land‐use composition: We expected trophic levels and habitat specialization to increase with the amount of habitat in the landscape and land‐use heterogeneity to reduce specialization, but increase richness and abundance. We observed that specialization and trophic levels increased with the amount of habitat in the landscape in farmlands and woodlands, but not in urban habitats. We observed an increase in richness with land‐use heterogeneity for all of the habitats except for woodlands. An increase in abundance with heterogeneity was only detected in farmlands. As expected, land‐use heterogeneity strongly and systematically reduced habitat specialization (Appendices 7 and 8).

## 4 DISCUSSION

Quantifying the response of biodiversity to land use and intensity in the context of the current biodiversity crisis is of paramount importance (Newbold et al., 2015). To our knowledge, our study is the first to (1) quantify the relative contributions of the land‐use composition and intensity to biological community patterns at the landscape scale using NPP_remaining_ and (2) show that the species–energy relationship also applies to the species–diversity relationship with NPP after human appropriation (NPP_remaining_), while accounting for confounding land use factors. Previous studies exploring the relative importance of the land‐use composition and intensity either have not accounted for the human impact on the availability of productivity (Duro et al., 2014; Hurlbert & Jetz, 2010) or have only accounted for a reduced set of land‐use variables (Haberl et al., 2005).

Employing a productivity measure that does not account for the proportion of productivity removed from the system by human uses may introduce some confusion into the interpretation of the results. Indeed, these variables provide information about the productivity generated by the ecosystem (NPP_actual_) but do not provide information about the available productivity of the ecosystem, which is assumed to drive community structure (Haberl et al., 2007). Therefore, NPP_remaining_ should be understood and employed as an indicator of the reverse intensity of human pressures on the ecosystem, and NPP should be used as an indicator of the ability of a system to produce biomass (Erb et al., 2013).

### 4.1 Relative importance of the composition and intensity of human pressures

In our study, land‐use composition and intensity variables primarily affected specialization and richness, with a more limited impact on trophic levels and abundance. The importance of the land‐use intensity was slightly dominant compared with the composition for richness, specialization, and trophic levels in farmland and urban areas, while the land‐use composition was a stronger predictor in woodlands and for abundance in general. Our findings regarding the importance of NPP_remaining_ for richness underpin the results of previous studies that have only accounted for land‐use heterogeneity and not land‐use proportions (Haberl et al., 2005; Hurlbert & Jetz, 2010) or have only focused on agricultural areas using a measure of productivity not accounting for harvesting (Duro et al., 2014). These three studies identified a better predictive power of productivity compared with land use, contrary to the findings of Coops, Wulder, and Iwanicka (2009), who studied the respective contributions of productivity (without accounting for harvesting and using fPAR‐derived variables) and land use (detailed variables). Our results highlight the importance of accounting for the land‐use composition while estimating the importance of productivity. The proportion of land use, which can be employed as a proxy of the habitat, but also of land‐use type and the spatial extent of human pressure, captures many different processes linked to the treatments (disturbance regimes) applied to the land, such as land management practices and human visits and activities. This strong synthetic characteristic of the land‐use composition can explain its importance for ecological processes.

NPP_remaining_ provides insights into the intensity of productivity appropriation that is not captured by land‐use variables and appears to have a significant impact on bird communities. The independent importance of the land‐use composition and intensity for bird communities has been previously observed in particular habitats, such as farmlands, for both specialization and trophic levels (Jeliazkov et al., 2016), or woodlands, for specialization (Drapeau et al., 2000). Our study generalizes these findings to three different habitats plus the landscape scale (the All‐habitats model) and to four community indices.

#### 4.1.1 Importance of NPP_remaining_ (intensity)

To our knowledge, only a few studies have investigated the link between NPP_remaining_ and species richness (Haberl et al., 2004, 2005; Mouchet et al., 2015). Consistent with Haberl et al. (2004, 2005), we found a positive relationship between the remaining productivity and species richness in our All‐habitats model, which suggest that natural and anthropogenic gradients of productivity tend to have similar effects on species richness. This result means that richness usually increases among farmlands, urban areas, and woodlands following the amount of NPP_remaining_ under these land uses.

However, among the individual habitat models, our results supported this relationship only in urban habitats. The variation of NPP_remaining_ was large within the three habitat types (Figure 2), and our results for farmlands and woodlands, therefore, cannot be explained by a smaller gradient of NPP_remaining_ in farmlands and woodlands than in urban areas. The species richness in farmlands appeared to be dependent on the land‐use composition, tending to be more closely linked to the area of farmland and land‐use heterogeneity than to the remaining productivity itself. This result shows that the nature of the heavy human pressures linked to the farming land use (recurrent, strong, and diversified disturbances) acts as a stronger driver of species richness than the available resources in farmland communities. Moreover, farmland specialists are adapted to low‐resource environments, and we showed that they are not favored by increased NPP_remaining_. Thus, it is the heterogeneity of NPP_remaining_ and of other habitats in the landscape that appears to increase the richness observed in farmland habitats (Filippi‐Codaccioni, Devictor, Bas, & Julliard, 2010; Jeliazkov et al., 2016). Species richness in woodlands was not linked to any composition or intensity variable. Further research would be needed to understand this result.

Our results also provide the first evidence that the remaining productivity is primarily linked to community specialization and much less to abundance, providing no support for the more individuals hypothesis in this context (Wright, 1983). We expected that more‐specialized communities would be found in low‐intensity areas with high remaining productivity because of fewer anthropogenic disturbances and/or more available resources (Abrams, 1995; Srivastava & Lawton, 1998). This relationship was only observed in woodlands, which represented the most natural habitat type included in this study. On the contrary, specialization in the Urban and All‐habitats models decreased with the remaining productivity. The results of the All‐habitats model can be explained by the higher specialization level of farmland‐specialist species than that of woodland‐specialist species, which increases specialization in low‐productivity areas of the study region. The negative relationship observed for urban communities may be explained by the nature of the urban habitat. Urban specialists are species that have to tolerate and are adapted to low available primary productivity in environments that are highly disturbed by diverse human activities. In such disturbed environments, the increase in available productivity is likely to benefit to generalist species, whose arrival would mathematically decreases the specialization of the community.

We did find support for the hypothesis that areas with high remaining productivity promote a high trophic level (Srivastava & Lawton, 1998). The effects of NPP_remaining_ on trophic level were observed in all of the habitats except for woodlands.

As expected for the four community indices, the importance of the remaining productivity differed among habitat types and was greater in highly anthropogenic habitats (Hurlbert, 2004). Our results indicated that the fastest increases in the various community indices with productivity occurred in the less‐productive habitats (urban and farmland areas), suggesting an increased importance of the available productivity when resources are scarce. It could also indicate a response to stronger gradients in vegetation complexity and biotic resource diversity in farmland and urban areas than those in woodlands. This result is consistent with previous macroecological findings showing that the dependence of birds (Hawkins, Field, & Cornell, 2003) on the available productivity increases at a global scale at higher latitudes, which are associated with lower levels of productivity (Phillips, Hansen, & Flather, 2008).

#### 4.1.2 Remarks on the importance of the heterogeneity of composition, land use, and NPP_remaining_

Our predictions regarding the responses of the community indices to the land‐use composition were generally validated by our results, confirming the importance of the area of habitat on the trophic levels and specialization of communities, independent of NPP_remaining_ (Allouche et al., 2012; Jeliazkov et al., 2016).

Both types of heterogeneity were important predictors of the community indices. These results emphasize the importance of the heterogeneity of the land‐use composition and intensity to the understanding of community responses to human pressures (Bohning‐Gaese, 1997; Hurlbert, 2004); however, they also highlight the importance of homogeneity of both the composition and intensity in promoting the habitat specialization of communities.

## 5 CONCLUDING REMARKS

We showed for the first time that the remaining productivity available to animals in human‐dominated ecosystems is an important driver of animal community patterns. Richness and habitat specialization appeared to be especially sensitive to the spatial variations of productivity. Land‐use composition variables (proportion and heterogeneity) were also important predictors of the community structure, thus demonstrating the importance of land‐use types in synthesizing human pressures and habitat types.

Land‐use intensity is expected to increase in the future to meet global food demands and may become the main driver of land use (Tilman, Balzer, Hill, & Befort, 2011); therefore, accounting for its impact on biodiversity is of primary importance. NPP_remaining_ appears to be a valuable indicator of the intensity of human pressures, complementary to the land‐use composition, providing important insights for all habitats types. Because this indicator directly refers to the productivity available for ecosystem functioning, it provides more valuable information than NPP_actual_, which is the metric that is usually used in studies of animal community patterns in anthropogenic landscapes. However, land‐use intensity is not unidimensional (Erb et al., 2013), and NPP_remaining_ may not capture all intensity dimensions (such as the use of pesticides or the effect of tillage). Further work is needed to evaluate the power of NPP_remaining_ as a synthetic indicator for the multidimensional aspects of intensity in relation to community patterns, which should also account for the annual temporal variation of NPP_remaining_ and explore its links with temporal changes in community structure.

## CONFLICT OF INTEREST

None declared.

## DATA ACCESSIBILITY

All of the data used for the analyses will be freely available via download on Dryad. These data include the tables used for richness, abundance, CTI, CSI, NPP_remaining_, and the standard deviation of NPP_remaining_ as well as the farmland, proportions of forest and urban areas, land‐use heterogeneity, and the spatial coordinates of each point. Please note that the provided community indices are the averaged values for the available years for each point. The raster of NPP_remaining_ covering the whole region is also available this archive site.

## AUTHOR CONTRIBUTIONS

VP, AM, and DC conceived the study concepts; VP and AM designed the methodology; VP collected the data and calculated NPP_remaining_ and biodiversity indices; AM and VP analyzed the data; and AM and VP led the writing of the manuscript. All authors contributed critically to the drafts and provided final approval for publication.

## APPENDIX 1

### 1.1

Monitoring surveys are sensitive to detectability issues, due to difference in the detection probability across habitats or throughout the year or a day. While there are several methods that allow correction of these variations in detectability over a large scale—for instance, to estimate population sizes—there are no methods allowing such correction if the desirable quantity is a measure of abundance at the count point scale (Bas, Devictor, Moussus, & Jiguet, 2008), which is our measure of interest here. To correct for detectability, one might add covariates to the model, where the main drawback would be that a covariate should not be correlated with the ecological processes under scrutiny (Bas et al., 2008). We, therefore, added two fixed effect covariates to the model: the Julian day and the time after sunset, both of which can influence the probability of an individual being detected, while not influencing its actual presence. We also added the local habitat as a random effect because the structure of the local habitat can influence the detection probability. Here, we argue that adding the local habitat as a random effect allows us to integrate the variability in the estimation of abundance due to habitat structure without influencing the outcome of our analyses because the local habitat is measured at a smaller scale than the process of interest (100 vs. 500 m).

## APPENDIX 2

### 2.1

The model‐averaging procedure consisted of (1) running every possible model—i.e.*,* every model nested within the model described in Equation (3); (2) selecting the best models based on the Akaike Information Criterion (AIC), keeping a delta‐AIC < 4 for all of the models; and (3) finally computing the weighted average of each coefficient, using the AIC weight to weight the average. We performed the model‐averaging procedure with the MuMIn package (Barton, 2013).

## APPENDIX 3

### 3.1

Results of analyses while running models built on the initial composition variables (PC1 and PC2) instead of their residuals (“Classic” series of models):

(a) *Results of hierarchical variance partitioning analyses*

Table A3 b) Coefficients and importance of each variable extracted from the model‐averaging procedure

**Table:**

		PC1	PC2	Land cover number	Mean NPP_remaining_	*SD* NPP_remaining_	Julian days	Minutes after sunset
Abundance	All habitats	**0.21 ± 0.02*** (1)**	0 ± 0.02 (0.21)	0.01 ± 0.02 (0.32)	0 ± 0.02 (0.2)	**0.04 ± 0.02** † **(0.8)**	**0.05 ± 0.01*** (1)**	**−0.05 ± 0.01*** (1)**
Abundance	Farmland	0.07 ± 0.04 (0.55)	−0.07 ± 0.04 (0.58)	**0.09 ± 0.04* (0.87)**	0.03 ± 0.05 (0.36)	0.05 ± 0.05 (0.43)	**0.06 ± 0.02** (0.98)**	0 ± 0.03 (0.27)
Abundance	Woodland	0.03 ± 0.02 (0.3)	0.02 ± 0.02 (0.22)	0.01 ± 0.02 (0.21)	0 ± 0.02 (0.18)	0.01 ± 0.02 (0.19)	**0.02 ± 0.01** † **(0.79)**	**−0.05 ± 0.02** (1)**
Abundance	Urban	**0.07 ± 0.04** † **(0.66)**	0 ± 0.04 (0.25)	−0.03 ± 0.04 (0.29)	−0.03 ± 0.04 (0.26)	−0.06 ± 0.04 (0.45)	**0.05 ± 0.02** (1)**	**−0.07 ± 0.02** (1)**
Species richness	All habitats	−0.02 ± 0.01 (0.39)	**−0.04 ± 0.01** (1)**	**0.07 ± 0.01*** (1)**	**0.05 ± 0.02*** (1)**	**0.08 ± 0.01*** (1)**	0.01 ± 0.01 (0.52)	**−0.05 ± 0.01*** (1)**
Species richness	Farmland	0.04 ± 0.03 (0.56)	**−0.05 ± 0.03** † **(0.68)**	**0.08 ± 0.03** (0.97)**	0.01 ± 0.03 (0.3)	**0.11 ± 0.04* (1)**	0.01 ± 0.01 (0.43)	**−0.03 ± 0.02* (0.76)**
Species richness	Woodland	0 ± 0.02 (0.19)	0.02 ± 0.02 (0.29)	0.01 ± 0.02 (0.19)	−0.01 ± 0.02 (0.19)	0.02 ± 0.02 (0.26)	0 ± 0.01 (0.19)	**−0.04 ± 0.01** (1)**
Species richness	Urban	0.02 ± 0.03 (0.28)	0 ± 0.03 (0.21)	**0.08 ± 0.03** (1)**	**0.09 ± 0.03** (1)**	0.04 ± 0.03 (0.38)	0.01 ± 0.01 (0.32)	**−0.08 ± 0.02*** (1)**
CTI	All habitats	**−0.03 ± 0.01*** (1)**	**−0.01 ± 0.01** † **(0.72)**	0.01 ± 0 (0.58)	**0.01 ± 0.01* (0.85)**	0.01 ± 0 (0.58)	**0.01 ± 0** (1)**	**0.01 ± 0* (0.92)**
CTI	Farmland	**−0.01 ± 0.01* (0.87)**	**−0.02 ± 0.01** † **(0.75)**	0 ± 0.01 (0.29)	0.01 ± 0.01 (0.49)	0 ± 0.01 (0.3)	−0.01 ± 0 (0.43)	**0.01 ± 0.01* (0.85)**
CTI	Woodland	0 ± 0 (0.19)	**−0.01 ± 0* (0.92)**	0 ± 0 (0.4)	0 ± 0 (0.21)	0 ± 0.01 (0.26)	**−0.01 ± 0** (1)**	0 ± 0 (0.33)
CTI	Urban	−0.01 ± 0.01 (0.41)	0 ± 0.01 (0.33)	0.01 ± 0.01 (0.64)	**0.02 ± 0.01** † **(0.81)**	0.01 ± 0.01 (0.55)	**0.04 ± 0*** (1)**	0 ± 0.01 (0.29)
CSI	All habitats	**0.12 ± 0.01*** (1)**	**0.05 ± 0.01*** (1)**	**−0.06 ± 0.01*** (1)**	**−0.05 ± 0.01*** (1)**	**−0.07 ± 0.01*** (1)**	**0.02 ± 0.01* (0.89)**	**0.02 ± 0.01** † **(0.68)**
CSI	Farmland	**−0.07 ± 0.03* (0.9)**	**0.04 ± 0.03** † **(0.63)**	**−0.04 ± 0.02** † **(0.61)**	−0.02 ± 0.03 (0.33)	**−0.13 ± 0.05* (1)**	**−0.03 ± 0.01* (0.9)**	0.02 ± 0.02 (0.39)
CSI	Woodland	**−0.03 ± 0.01* (1)**	**−0.04 ± 0.01** (1)**	**−0.03 ± 0.01* (0.91)**	**−0.02 ± 0.01* (0.79)**	0 ± 0.01 (0.18)	−0.01 ± 0.01 (0.39)	0 ± 0.01 (0.18)
CSI	Urban	0.02 ± 0.02 (0.39)	0.03 ± 0.03 (0.48)	**−0.07 ± 0.03** (1)**	**−0.05 ± 0.02* (0.79)**	**−0.07 ± 0.03* (1)**	**0.03 ± 0.01** (1)**	**0.04 ± 0.02** (1)**

The importance value is provided in parentheses. An importance of 0.5 corresponds to the selection of the variable in 50% of the best models (delta‐AIC = 4), and an importance of 1 corresponds to the selection of the variable in 100% of the best models. Bold values indicate p < 0.1.

^†^ *p* < .1; **p* < .05; ***p* < .01; ****p* < .001.

## APPENDIX 4

### 4.1

Results of analyses while running models built on the residuals of NPP_remaining_ regressed on the composition variables PC1 and PC2 (“Residual NPP_remaining_” series of models):

*(a) Results of the hierarchical variance partitioning analyses*

Table A4  b) Coefficients and importance of each variable extracted from the model‐averaging procedure

**Table:**

		PC1	PC2	Land cover number	Mean NPPt	*SD* NPPt	Julian days	Minutes after sunset
Abundance	All habitats	**0.21 ± 0.02** *** **(1)**	0 ± 0.02 (0.25)	0.03 ± 0.02 (0.5)	0 ± 0.02 (0.24)	0.03 ± 0.02 (0.58)	**0.05 ± 0.01** *** **(1)**	**−0.05 ± 0.01** *** **(1)**
Abundance	Farmland	0.06 ± 0.04 (0.5)	**−0.08 ± 0.04** † **(0.67)**	**0.1 ± 0.04** ** **(0.94)**	0.01 ± 0.04 (0.28)	0.05 ± 0.05 (0.44)	**0.06 ± 0.02** ** **(0.98)**	0 ± 0.03 (0.27)
Abundance	Woodland	0.03 ± 0.02 (0.3)	0.02 ± 0.03 (0.22)	0.02 ± 0.02 (0.22)	0.01 ± 0.02 (0.19)	0 ± 0.02 (0.18)	**0.02 ± 0.01** † **(0.79)**	**−0.05 ± 0.02** ** **(1)**
Abundance	Urban	**0.09 ± 0.04** * **(0.87)**	0.02 ± 0.04 (0.21)	−0.05 ± 0.05 (0.37)	−0.03 ± 0.03 (0.24)	−0.02 ± 0.04 (0.27)	**0.05 ± 0.02** ** **(1)**	**−0.08 ± 0.03** ** **(1)**
Species richness	All habitats	**−0.06 ± 0.01** *** **(1)**	**−0.13 ± 0.01** *** **(1)**	**0.15 ± 0.01** *** **(1)**	**0.05 ± 0.01** ** **(1)**	**0.08 ± 0.01** *** **(1)**	0.01 ± 0.01 (0.54)	**−0.05 ± 0.01** *** **(1)**
Species richness	Farmland	0.03 ± 0.03 (0.45)	**−0.05 ± 0.03** † **(0.72)**	**0.12 ± 0.03** *** **(1)**	0 ± 0.02 (0.27)	**0.11 ± 0.05** * **(1)**	0.01 ± 0.01 (0.43)	**−0.03 ± 0.02** * **(0.76)**
Species richness	Woodland	0 ± 0.02 (0.17)	0.02 ± 0.02 (0.31)	0.01 ± 0.02 (0.21)	0 ± 0.02 (0.16)	0.01 ± 0.02 (0.24)	0 ± 0.01 (0.2)	**−0.04 ± 0.01** ** **(1)**
Species richness	Urban	0.02 ± 0.05 (0.26)	−0.03 ± 0.03 (0.34)	**0.2 ± 0.04** *** **(1)**	**0.1 ± 0.03** ** **(1)**	0.04 ± 0.04 (0.44)	0.01 ± 0.01 (0.33)	**−0.08 ± 0.02** *** **(1)**
CTI	All habitats	**−0.04 ± 0.01** *** **(1)**	**−0.03 ± 0.01** *** **(1)**	**0.01 ± 0** ** **(1)**	**0.01 ± 0** * **(0.75)**	0 ± 0 (0.5)	**0.01 ± 0** ** **(1)**	**0.01 ± 0** * **(0.9)**
CTI	Farmland	**−0.01 ± 0.01** ** **(0.91)**	**−0.02 ± 0.01** ** **(0.96)**	0 ± 0.01 (0.28)	0 ± 0.01 (0.35)	0 ± 0.01 (0.31)	−0.01 ± 0 (0.43)	**0.01 ± 0.01** * **(0.85)**
CTI	Woodland	0 ± 0 (0.16)	**−0.01 ± 0** * **(0.94)**	−0.01 ± 0 (0.45)	0 ± 0 (0.17)	0 ± 0 (0.2)	**−0.01 ± 0** ** **(1)**	0 ± 0 (0.37)
CTI	Urban	−0.01 ± 0.01 (0.39)	−0.01 ± 0.01 (0.45)	**0.04 ± 0.01** ** **(1)**	0.01 ± 0.01 (0.55)	**0.01 ± 0.01** † **(0.61)**	**0.04 ± 0** *** **(1)**	0 ± 0.01 (0.31)
CSI	All habitats	**0.18 ± 0.01** *** **(1)**	**0.14 ± 0.01** *** **(1)**	**−0.12 ± 0.01** *** **(1)**	**−0.05 ± 0.01** *** **(1)**	**−0.06 ± 0.01** *** **(1)**	**0.02 ± 0.01** * **(0.89)**	**0.02 ± 0.01** † **(0.68)**
CSI	Farmland	**−0.05 ± 0.03** † **(0.72)**	**0.05 ± 0.03** * **(0.77)**	**−0.1 ± 0.02** *** **(1)**	−0.01 ± 0.02 (0.28)	**−0.12 ± 0.05** * **(1)**	**−0.03 ± 0.01** ** **(0.91)**	0.02 ± 0.02 (0.39)
CSI	Woodland	**−0.03 ± 0.01** * **(0.96)**	**−0.03 ± 0.01** * **(0.86)**	**−0.03 ± 0.01** * **(1)**	**−0.03 ± 0.01** * **(0.81)**	−0.01 ± 0.01 (0.25)	−0.01 ± 0.01 (0.35)	0 ± 0.01 (0.16)
CSI	Urban	**0.08 ± 0.04** † **(0.67)**	**0.06 ± 0.03** * **(0.77)**	**−0.23 ± 0.05** *** **(1)**	**−0.05 ± 0.03** † **(0.61)**	**−0.08 ± 0.03** ** **(0.96)**	**0.04 ± 0.01** ** **(1)**	**0.05 ± 0.02** ** **(1)**

The importance value is provided in parentheses. An importance of 0.5 corresponds to the selection of the variable in 50% of the best models (delta‐AIC = 4), and an importance of 1 corresponds to the selection of the variable in 100% of the best models. Bold values indicate p < 0.1.

^†^ *p* < .1; **p* < .05; ***p* < .01; ****p* < .001.

## APPENDIX 5

### 5.1

Plot of the variations in the community indices with Res(PC1) for the four models (All‐habitats, Farmland, Urban, and Woodland) from the “Residual composition” set of models. The dots represent the points, and the lines represent the relationships obtained with the models. The error on either side of the lines corresponds to the standard error.

## APPENDIX 6

### 6.1

Plot of the variations in the community indices with Res(PC2) for the four models (All‐habitats, Farmland, Urban, and Woodland) from the “Residual composition” set of models. The dots represent the points, and the lines represent the relationships obtained with the models. The error on either side of the lines corresponds to the standard error.

## APPENDIX 7

### 7.1

Plot of the variations of the community indices with the number of land uses for the four models (All‐habitats, Farmland, Urban, and Woodland) from the “Residual composition” set of models. The dots represent the points, and the lines represent the relationships obtained with the models. The error on either side of the lines corresponds to the standard error.

## APPENDIX 8

### 8.1

Plot of the variations of the community indices with the standard deviation of NPP_remaining_ for the four models (All‐habitats, Farmland, Urban, and Woodland) from the “Residual composition” set of models. The dots represent the points, and the lines represent the relationships obtained with the models. The error on either side of the lines corresponds to the standard error.
